# Broader and safer clinically-relevant activities of pentadecanoic acid compared to omega-3: Evaluation of an emerging essential fatty acid across twelve primary human cell-based disease systems

**DOI:** 10.1371/journal.pone.0268778

**Published:** 2022-05-26

**Authors:** Stephanie K. Venn-Watson, Camden N. Butterworth

**Affiliations:** 1 Epitracker, Inc., San Diego, California, United States of America; 2 Seraphina Therapeutics, Inc., San Diego, California, United States of America; 3 Texas Christian University, Fort Worth, Texas, United States of America; University of Alberta, CANADA

## Abstract

A growing body of evidence supports that pentadecanoic acid (C15:0), an odd-chain saturated fat found in butter, is an essential fatty acid that is necessary in the diet to support long-term metabolic and heart health. Here, dose dependent and clinically relevant cell-based activities of pure C15:0 (FA15^TM^) were compared to eicosapentaenoic acid (EPA), a leading omega-3 fatty acid, as well as to an additional 4,500 compounds. These studies included 148 clinically relevant biomarkers measured across 12 primary human cell systems, mimicking various disease states, that were treated with C15:0 at four different concentrations (1.9 to 50 μM) and compared to non-treated control systems. C15:0 was non-cytotoxic at all concentrations and had dose dependent, broad anti-inflammatory and antiproliferative activities involving 36 biomarkers across 10 systems. In contrast, EPA was cytotoxic to four cell systems at 50 μM. While 12 clinically relevant activities were shared between C15:0 and EPA at 17 μM, C15:0 had an additional 28 clinically relevant activities, especially anti-inflammatory, that were not present in EPA. Further, at 1.9 and 5.6 μM, C15:0 had cell-based properties similar to bupropion (Pearson’s scores of 0.78), a compound commonly used to treat depression and other mood disorders. At 5.6 μM, C15:0 mimicked two antimicrobials, climabazole and clarithromycin (Pearson’s scores of 0.76 and 0.75, respectively), and at 50 μM, C15:0 activities matched that of two common anti-cancer therapeutics, gemcitabine and paclitaxel (Pearson’s scores of 0.77 and 0.74, respectively). In summary, C15:0 had dose-dependent and clinically relevant activities across numerous human cell-based systems that were broader and safer than EPA, and C15:0 activities paralleled common therapeutics for mood disorders, microbial infections, and cancer. These studies further support the emerging role of C15:0 as an essential fatty acid.

## Introduction

Fatty acids play major roles in supporting physiological health. This includes important basic roles, such as energy storage and transport, gene regulation, and thermal and electrical insulation in cells. Additionally, fatty acids act as building blocks for cell membranes, while facilitating cellular signaling [1]. Fatty acids can provide direct and broad health benefits, including anti-inflammatory, antitumor, antihypertensive, and antidiabetic activities [2].

Essential fatty acids are named as such because they cannot be efficiently made by the body yet are required to maintain our physiological health [3]. As such, adequate amounts of essential fatty acids must be ingested *via* food or supplements. The two best known essential fatty acids are α-linolenic acid, an omega-3 fatty acid, and linoleic acid, an omega-6 fatty acid [4]. Eicosapentaenoic acid (EPA) and docosahexaenoic acid (DHA) are the two most commonly known and studied omega-3 fatty acids; while they can be made by the body using α-linolenic acid, this conversion process is not necessarily efficient, increasing the need to acquire EPA and DHA directly from food or supplements [5].

Associations between essential fatty acids and lower risks of heart disease, type 2 diabetes, cancer, mood disorders, arthritis and neurological diseases have suggested their potential to prevent, manage and treat a wide variety of diseases [3, 6]. Anti-inflammatory properties of omega-3s may help people manage rheumatoid arthritis and other inflammatory conditions, such as asthma [7]. The pathway by which omega-3s are delivered to the brain supports their development as therapeutics for neurodegenerative disorders [8]. Further, the American Heart Association recommends EPA and DHA supplements to patients diagnosed with heart disease and hypertriglyceridemia [9]. Currently in the United States, Lovaza^®^ and Vascepa^®^ are two omega-3 prescription therapeutics that are FDA approved to lower triglyceride levels and improve the condition of high-risk cardiovascular patients [6]. An extensive clinical trial has shown that Vascepa^®^, which contains pure EPA (icosapent ethyl EPA), successfully reduced the risk of major ischemic events compared to placebo [10]. This outcome was not found in two similar large clinical trials involving prescription omega-3 products containing both EPA and DHA [11, 12]. As a result, there has been increasing interest in understanding the potential added benefits of pure EPA products [13].

Unlike omega-3s, which are polyunsaturated fatty acids, pentadecanoic acid (C15:0) is an odd-chain saturated fatty acid present in trace levels in dairy fat and ruminant meat, as well as some types of plants and fish [14]. Large, prospective human cohort studies have shown that higher C15:0 blood concentrations are associated with lower risks of developing chronic conditions over time, including type 2 diabetes, cardiovascular disease, and heart failure [15–17]. Higher dietary intake and circulating concentrations of C15:0 have also been linked to lower mortality and greater longevity, as well as to lower risks of chronic inflammation, gestational diabetes, hypertension, nonalcoholic fatty liver disease, as well as less severe nonalcoholic steatohepatitis and chronic obstructive pulmonary disease [17–25].

Beyond population-based studies, experimental research has shown that C15:0 is an active and beneficial fatty acid with direct pleiotropic activities relevant to stemming chronic conditions, especially with age [26–29]. Specifically, C15:0 is a dual partial peroxisome proliferator-activated receptor α/δ agonist, AMP-activated protein kinase activator, and histone deacetylase 6 inhibitor [26–28]. Further, C15:0 has been shown to repair mitochondrial function, improve the stability of red blood cells, regulate glucose metabolism, and decrease proliferation of cancer cells [26–29].

These pleiotropic cell-based activities likely explain C15:0’s clinically-relevant benefits that have been observed *in vivo*. High fat diet-induced obese mice supplemented daily with oral C15:0 for 12 weeks had lower glucose, cholesterol, body weight gain, and pro-inflammatory cytokines compared to non-supplemented controls [28]. Daily C15:0 supplementation lowered inflammation, cholesterol, and triglycerides, as well as attenuated anemia and liver fibrosis, in a model of nonalcoholic fatty liver disease [28]. Because C15:0 is an established dietary active fatty acid not readily made by the body, has lower body levels associated with poorer cardiometabolic and liver health, and has demonstrated beneficial and pleiotropic activities directly related to cardiometabolic, immune and liver health, C15:0 has been proposed as an essential fatty acid [28, 30].

Here, we hypothesized and demonstrated that C15:0’s broad cell-based and clinically relevant activities would 1) match or exceed that of EPA, a leading omega-3 fatty acid and FDA-approved prescription therapeutic with demonstrated efficacy in successfully reducing the risk of major cardiovascular events, as well as 2) share clinically relevant properties with well-established therapeutic compounds.

## Materials and methods

### C15:0 compounds

To evaluate the repeatability and consistency of human cell phenotypic profiling with C15:0, two C15:0 compounds manufactured at different facilities, Seraphina Therapeutics (FA15^TM^, pentadecanoic acid 99%) and Millipore Sigma (Sigma-Aldrich Product W433400, pentadecanoic acid 99%), were compared using the BioMAP^®^ Diversity Plus Panel (Eurofins DiscoverX, Fremont, California USA).

### BioMAP® Diversity PLUS panel

The BioMAP^®^ Diversity PLUS panel (Eurofins DiscoverX, Fremont, California USA) uses 12 human primary cell-based systems designed to model complex human tissue and disease biology of the vasculature, skin, lung, and inflammatory tissues. Quantitative measurements of 148 clinically relevant biomarker activities across this broad panel, along with comparative analysis of the biological activities of known bioactive agents in the BioMAP reference database, are used to predict the safety, efficacy and function of test agents. The reference database includes BioMAP profiles of over 4,500 compounds with known safety, efficacy and function.

#### Cell exposure studies

Human primary cells in Eurofins’ BioMAP systems were used at early passage (passage 4 or earlier) to minimize adaptation to cell culture conditions and preserve physiological signaling responses. All cells, including human umbilical vein endothelial cells (HUVEC), peripheral blood mononuclear cells (PBMC), CD19 positive B cells, bronchial epithelial cells, coronary artery smooth muscle cells, human dermal fibroblasts neonatal (HDFn), keratinocytes, differentiated lung myofibroblasts and M1 macrophages, were each from a pool of multiple healthy human donors (n = 2 to 6), commercially purchased and handled according to the recommendations of the manufacturers. The following vendors supplied Eurofins DiscoverX with primary human cells, all of which followed ethical guidelines and documented informed consent: Cell Applications, Inc., CellzDirect, Celsis-IVT, Leukolab, Life Technologies, Lonza, ScienCell, Hemacare Corporation, Stemcell Technologies, AllCells, Physician’s Plasma Alliance, Lifeline Cell Technologies, and Zen-Bio, Inc. Human blood derived CD14+ monocytes were differentiated into macrophages *in vitro* before being added to the *l*Mphg system.

Cell types and stimuli used in each system were as follows: 3C system [HUVEC + *(IL-1β*, *TNFα and IFNγ)*], 4H system [HUVEC + *(IL-4 and histamine)*], LPS system [PBMC and HUVEC + *LPS (TLR4 ligand)*], SAg system [PBMC and HUVEC + *TCR ligands*], BT system [CD19+ B cells and PBMC + *(α-IgM and TCR ligands)*], BF4T system [bronchial epithelial cells and HDFn + *(TNFα and IL-4)*], BE3C system [bronchial epithelial cells + *(IL-1β*, *TNFα and IFNγ)*], CASM3C system [coronary artery smooth muscle cells + *(IL-1β*, *TNFα and IFNγ)*], HDF3CGF system [HDFn + *(IL-1β*, *TNFα*, *IFNγ*, *EGF*, *bFGF and PDGF-BB)*], KF3CT system [keratinocytes and HDFn + *(IL-1β*, *TNFα*, *IFNγ and TGFβ)*], MyoF system [differentiated lung myofibroblasts + *(TNFα and TGFβ)*] and *l*Mphg system [HUVEC and M1 macrophages + *Zymosan (TLR2 ligand)*]. Further information on the cell systems used, including biomarker readouts, are provided in S1 Table.

Systems were derived from either single cell types or co-culture systems. Adherent cell types were cultured in 96 or 384-well plates until confluence, followed by the addition of PBMC (SAg and LPS systems). The BT system consisted of CD19+ B cells co-cultured with PBMC and stimulated with a BCR activator and low levels of TCR stimulation. Test agents prepared in either DMSO (small molecules; final concentration ≤ 0.1%) or PBS (biologics) were added at the indicated concentrations 1-hr before stimulation and remained in culture for 24-hrs or as otherwise indicated (48-hrs, MyoF system; 72-hrs, BT system (soluble readouts); 168-hrs, BT system (secreted IgG)). Each plate contained drug controls (e.g., legacy control test agent colchicine at 1.1 μM), negative controls (e.g., non-stimulated conditions) and vehicle controls (e.g., 0.1% DMSO) appropriate for each system.

Direct ELISA was used to measure biomarker levels of cell-associated and cell membrane targets. Soluble factors from supernatants were quantified using either HTRF^®^ detection, bead-based multiplex immunoassay or capture ELISA. Overt adverse effects of test agents on cell proliferation and viability (cytotoxicity) were detected by sulforhodamine B (SRB) staining, for adherent cells, and alamarBlue® reduction for cells in suspension. For proliferation assays, individual cell types were cultured at subconfluence and measured at time points optimized for each system (48-hrs: 3C and CASM3C systems; 72-hrs: BT and HDF3CGF systems; 96-hrs: SAg system). Cytotoxicity for adherent cells was measured by SRB (24-hrs: 3C, 4H, LPS, SAg, BF4T, BE3C, CASM3C, HDF3CGF, KF3CT, and *l*Mphg systems; 48-hrs: MyoF system), and by alamarBlue staining for cells in suspension (24-hrs: SAg system; 42-hrs: BT system) at the time points indicated.

Inflammation-related biomarkers included the following: sPGE2, sTNFα, IL-6, ICAM, VCAM, E-selectin, P-selectin, IL-8, eotaxin-3, MCP-1, IP10, MIG, ITAC, SAA, MIP1α, and IL-1α. Immunomodulatory biomarkers included the following: CD69, SigG, CD38, CD40, HLA-DR, IL-2, and IL-6. Fibrosis-related biomarkers included the following: Col-I, Col-III, Col-IV, PAI-I, uPAR, uPA, tPA, MMP-1, MMP-3, MMP-9, TIMP-1, TIMP-2, Ker8/18, decorin, EGFR, αSMA, bFGF, and CD90. Hemostasis-related biomarkers included TF and TM. Additional biomarkers included VEGFR-2 and LDLR.

#### Profile analysis

Biomarker activities were annotated when 2 or more consecutive concentrations changed in the same direction relative to vehicle controls, were outside of the significance envelope and had at least one concentration with an effect size > 20% (|log_10_ *ratio*| > 0.1). Biomarker key activities were described as modulated if these activities increased in some systems but decreased in others. Cytotoxic conditions were noted when total protein levels decreased by more than 50% (log_10_ *ratio* of SRB or alamarBlue levels < -0.3) and were indicated by a thin black arrow above the X-axis. A compound was considered to have broad cytotoxicity when cytotoxicity was detected in 3 or more systems. Concentrations of test agents with detectable broad cytotoxicity were excluded from biomarker activity annotation and downstream benchmarking, similarity search and cluster analysis. Antiproliferative effects were defined by an SRB or alamarBlue log10 *ratio* value < -0.1 from cells plated at a lower density and were indicated by grey arrows above the X-axis. Cytotoxicity and antiproliferative arrows only required one concentration to meet the indicated threshold for profile annotation.

#### Compound comparisons

Activities between C15:0 from two different sources (Sigma Product W433400 and Seraphina Therapeutics FA15^TM^), as well as between FA15^TM^ and EPA, were considered common if a biomarker readout in a specific system was outside the significance envelope in the same direction and had an effect size > 20% (|log_10_ *ratio*| > 0.1). Activities were considered as differentiated if a biomarker readout in a specific system was outside the significance envelope for one compound but not the other; or if a biomarker readout in a specific system was outside the significance envelope in one direction for one compound and in the opposite direction for the other compound.

#### Broad similarity analysis

When comparing C15:0 with over 4,500 test agents in a central BioMAP database, common biomarker readouts were annotated when the readout for both profiles was outside of the significance envelope with an effect size > 20% in the same direction. Concentrations of test agents that had 3 or more detectable systems with cytotoxicity were excluded from similarity analysis. Concentrations of test agents that had 1–2 systems with detectable cytotoxicity were included in the similarity search analysis, along with an overlay of the database match with the top concentration of the test agent. This was followed by an additional overlay of the next highest concentration of the test agent containing no systems with detectable cytotoxicity and the respective database match.

To determine the extent of similarity between BioMAP profiles of compounds run in the Diversity PLUS panel, a custom similarity metric (BioMAP Z-Standard) was developed that was a combinatorial approach that had improved performance in mechanism classification of reference agents compared to other measures tested (including Pearson’s and Spearman’s correlation coefficients). This approach more effectively accounted for variations in the number of data points, systems, active biomarker readouts and the amplitude of biomarker readout changes that are characteristic features of BioMAP profiles. A Pearson’s correlation coefficient (*r*) was first generated to measure the linear association between two profiles that was based on the similarity in the direction and magnitude of the relationship. Since the Pearson’s correlation can be influenced by the magnitude of any biomarker activity, a per-system weighted average Tanimoto metric was used as a filter to account for underrepresentation of less robust systems. The Tanimoto metric did not consider the amplitude of biomarker activity but addressed whether the identity and number of readouts were in common on a weighted, per system basis. A real-value Tanimoto metric was calculated first by normalizing each profile to the unit vector. Then, it was incorporated into a system weighted-averaged real-value Tanimoto metric. The calculation used the real-value Tanimoto score for each *i*th system (T_i_) and the weight of each *i*th system (W_i_). W_i_ was calculated for each system.

Based on the optimal performance of reference compounds, profiles were identified as having mechanistically relevant similarity if the Pearson’s correlation coefficient (r) ≥ 0.7. Finally, a Fisher r-to-z-transformation was used to calculate a z-score to convert a short tail distribution into a normal distribution as follows: z = 0.5 log 1+r. Then the BioMAP Z-Standard, which adjusted for the number of common readouts (CR), was generated according to the 10 1−r √ following formula: Z-Standard = z · CR − 3. A larger BioMAP Z-Standard value corresponded to a higher confidence level, and this was the metric used to rank similarity results. Differentiating biomarkers are defined when one profile has a readout outside of the significance envelope with an effect size > 20% (|log10 *ratio*| > 0.1), and the readout for the other profile is either inside the envelope or in the opposite direction.

## Results

### Pentadecanoic acid compound comparisons

**Fig 1:**
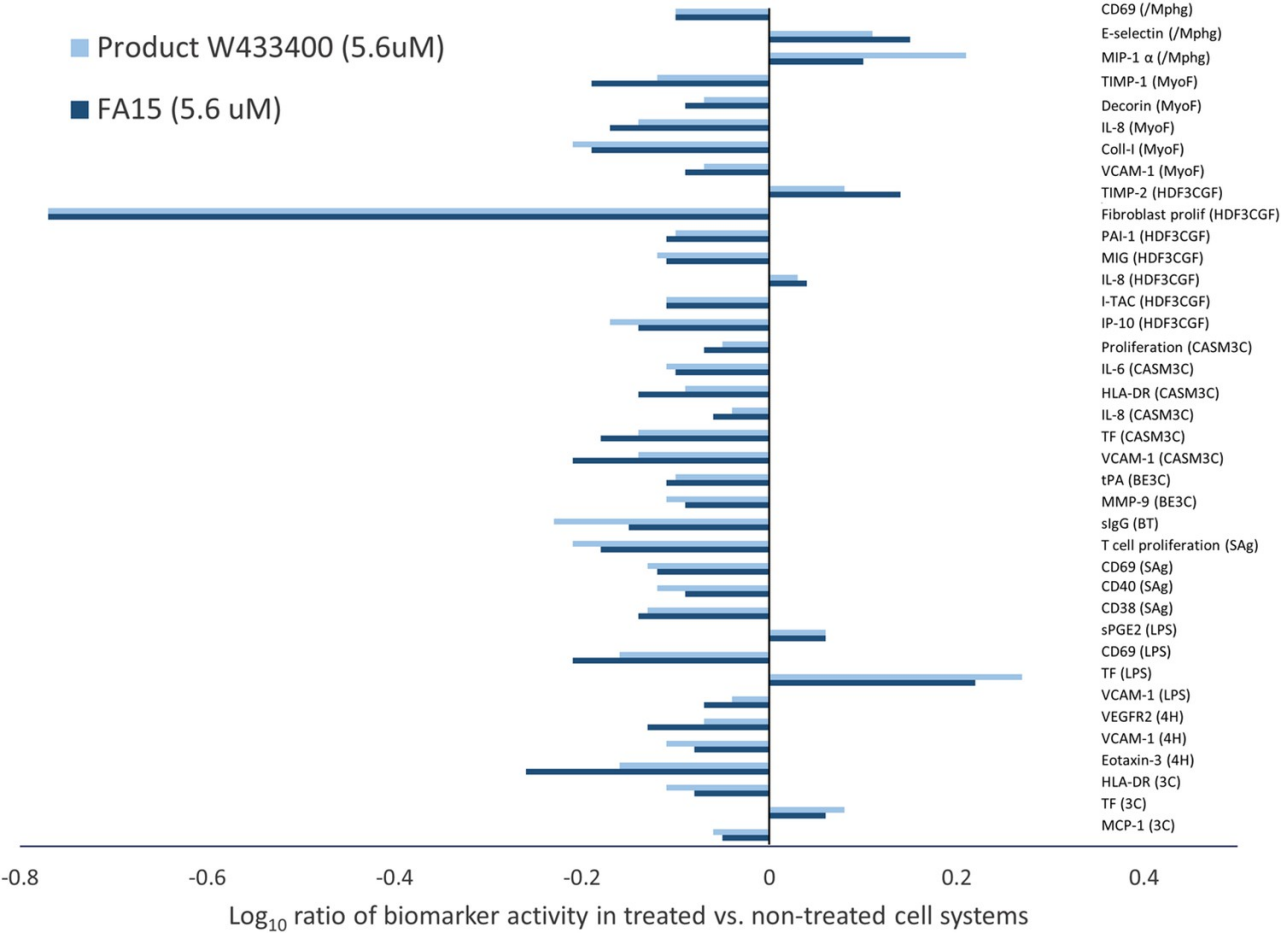
Shared human cell-based activities between two C15:0 compounds. Clinically relevant biomarker activities that were common when comparing two C15:0 compounds (> 98% purity, 5.6 μM) acquired from different manufacturers (Product W433400 from Sigma Aldrich and FA15^TM^ from Seraphina Therapeutics). Biomarker readouts were considered common when the readout for both profiles was outside the significance envelope in the same direction.

Expanded comparative overlay analysis of two free fatty acid C15:0 compounds (> 99%) acquired from Seraphina Therapeutics (FA15^TM^) and Sigma Aldrich (Product W433400) at 5.6 μM revealed 38 common activities among 11 of the 12 systems that were outside the significance envelope in the same direction (Fig 1). Both of the C15:0 compounds were non-cytotoxic at all concentrations.

Of the 38 common activities, 11 were inflammation-related, most of which were anti-inflammatory activities (lower IL-6, VCAM, e-selectin, eotaxin-3, MCP-1, IP10, MIG, and ITAC). In contrast, sPGE2 and MIP1α, were higher in both C15:0-treated panels compared to controls. While IL-8 was lower in two systems (MyoF and CASM3C) treated with either C15:0 compound, IL-8 was higher in a third system (HDF3CGF), supporting that C15:0’s effects on IL-8 differed based on the specific cell system treated.

Both C15:0 compounds down-regulated five activities related to immunomodulation (lower CD69, secreted IgG, CD38, HLA-DR, and IL-6) compared to non-treated control systems. Further, seven activities shared between both C15:0 compounds were related to fibrosis, most of which (collagen I, PAI-I, tPA, MMP-9, TIMP-1 and decorin) were lower compared to non-treated control systems. One fibrosis-related biomarker, TIMP-2, was higher in both C15:0-treated panels. One common biomarker for hemostasis, TF, was higher in two systems (LPS and 3C) and lower in a third system (CASM3C) compared to non-treated systems. An additional biomarker activity shared between both C15:0 compounds was lower VEGFR2.

**Fig 2:**
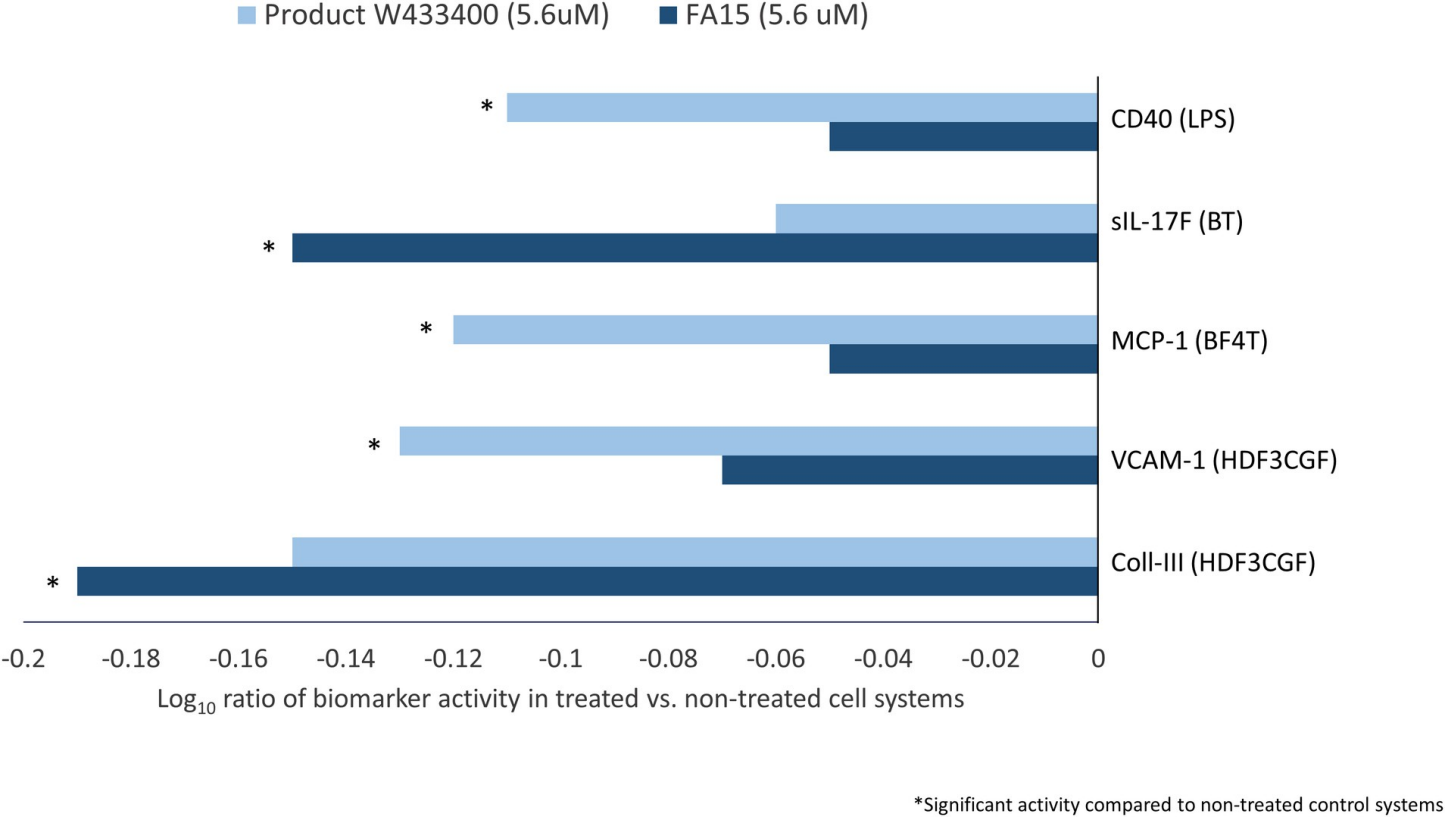
Differentiating human cell-based activities between two C15:0 compounds. Clinically relevant biomarker activities that were different when comparing two C15:0 compounds (> 98% purity, 5.6 μM) acquired from different manufacturers (Product W433400 from Sigma Aldrich and FA15^TM^ from Seraphina Therapeutics). Different biomarker readouts were included when the readout for one compound was outside the significance envelope with an effect size > 20% (|log_10_ *ratio*| > 0.1) in the same direction, and the other was not or was outside the significance envelope in the opposite direction.

Of the 148 biomarker readouts, the following five biomarker activities were not shared between the two C15:0 compounds: CD40, secreted IL-17F, MCP-1, VCAM-1, and collagen III (Fig 2). Specifically, the C15:0 Product W433400 from Sigma Aldrich significantly lowered CD40 in the LPS system, MCP-1 in the BF4T system, and VCAM-1 in the HDF3CGF in system, while FA15^TM^ trended in the same direction, but without reaching statistical significance. Conversely, FA15^TM^ significantly lowered sIL-17F in the BT system and collagen III in the HDF3CGF system, while Product W433400 from Sigma Aldrich trended in the same direction, but without reaching statistical significance.

Overall similarities in the two compounds’ phenotypic profiles support that C15:0 compounds meeting the same manufacturing criteria and purity specifications (> 98%) can achieve consistent and repeatable outcomes.

### Annotated, dose-dependent activities

**Fig 3:**
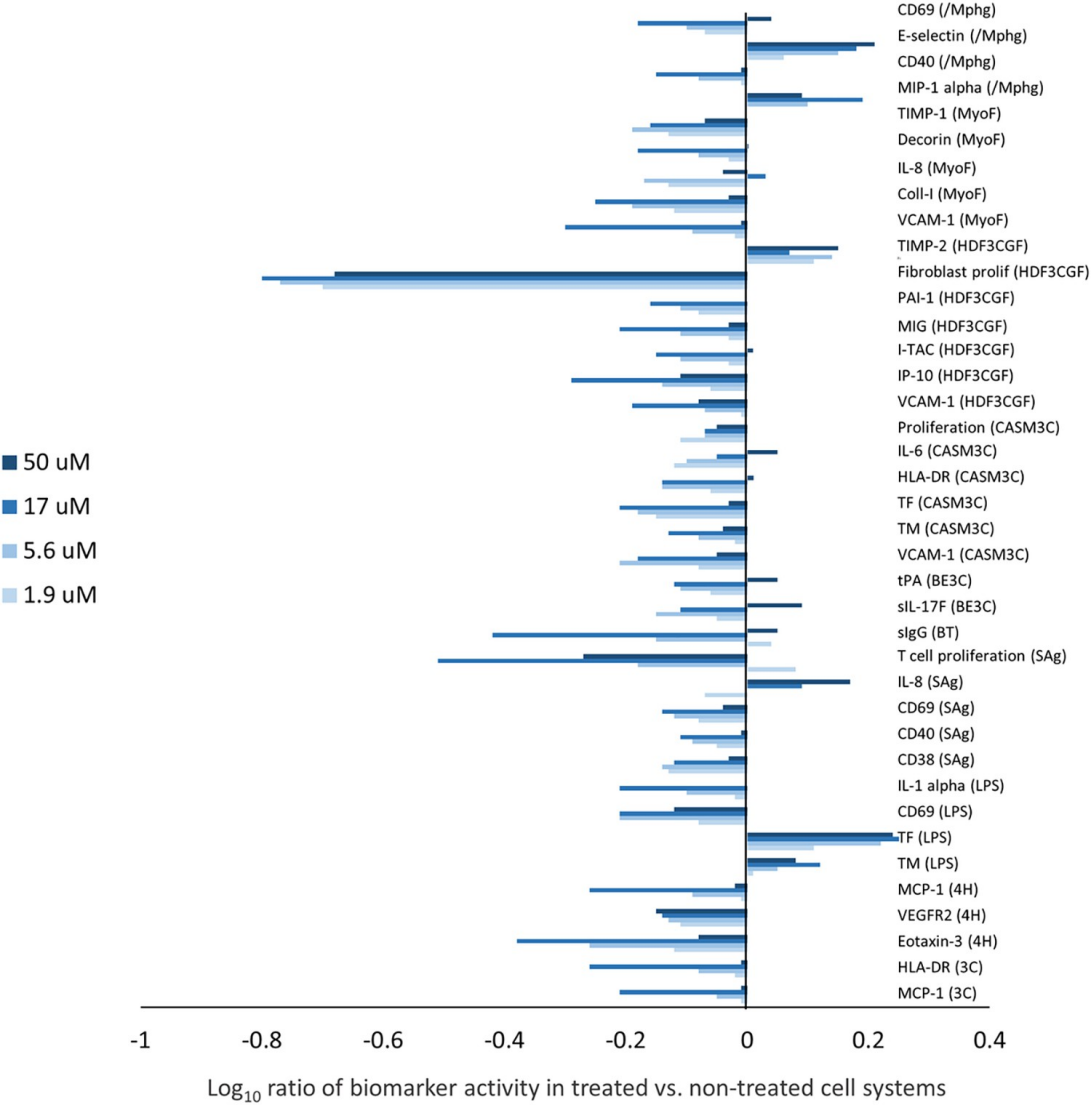
C15:0 annotated human cell-based activities. Clinically relevant and dose-dependent biomarker activities of C15:0 (> 98% purity, FA15^TM^) at four concentrations ranging from 1.9 to 50 μM. Biomarker activities were annotated when 2 or more consecutive concentrations changed in the same direction relative to vehicle controls, were outside of the significance envelope, and had at least one concentration with an effect size > 20% (|log_10_ *ratio*| > 0.1).

Given the demonstrated repeatable activities between the two C15:0 compounds, annotated, dose-dependent activities were determined for the C15:0 compound from Seraphina Therapeutics (FA15^TM^). C15:0 was non-cytotoxic across all 12 systems and all concentrations tested (1.9 to 50 μM). C15:0 was an active compound with 36 annotated and clinically relevant activities in 10 of the 12 systems in the BioMAP Diversity PLUS Panel (Fig 3). Specifically, C15:0 impacted multiple inflammation-related activities, most of which were anti-inflammatory and included decreased eotaxin 3, MCP-1, VCAM-1, I-TAC, MIG, IL-8, IP-10, IL-1α, and IL-6 levels. C15:0 was immune-inhibitory with lowered CD40, sIgG, HLA-DR, CD38, CD69, and sIL-17F levels detected compared to non-treated control systems. Additionally, C15:0 lowered several matrix and tissue remodeling activities in cell systems mimicking fibrotic diseases, including collagen I, decorin, TIMP-1, tPA, and PAI-1, and increased TIMP-2. C15:0 also modulated hemostasis activities (TF and TM), and decreased VEGFR2 levels. In cell-based systems mimicking cancer and fibrotic diseases, C15:0 was antiproliferative at the indicated concentrations: endothelial cells (50 μM, 17 μM), T cells (50 μM, 17 μM, 5.6 μM), coronary artery smooth muscle cells (1.9 μM), and fibroblasts (50 μM, 17 μM, 5.6 μM, 1.9 μM).

### Comparisons with eicosapentaenoic acid (EPA)

At 50 μM, EPA was cytotoxic to the following four of the 12 primary human cell systems: 3C (containing venular endothelial cells), BE3C (containing bronchial epithelial cells), HDF3GCF (containing dermal fibroblasts), and MyoF (containing lung fibroblasts). Overt adverse effects of compounds on viability (cytotoxicity) were detected by sulforhodamine B staining, for adherent cells (24 hours in the 3C, BE3C and HDF3GCF systems and 48 hours in the MyoF system), and alamarBlue^®^ reduction for cells in suspension. Cytotoxic conditions were noted when total protein levels decreased by more than 50% (log10 ratio of SRB or alamarBlue levels < -0.3). Broad cytotoxicity at a given concentration was defined when cytotoxicity was detected in 3 or more systems.

**Fig 4:**
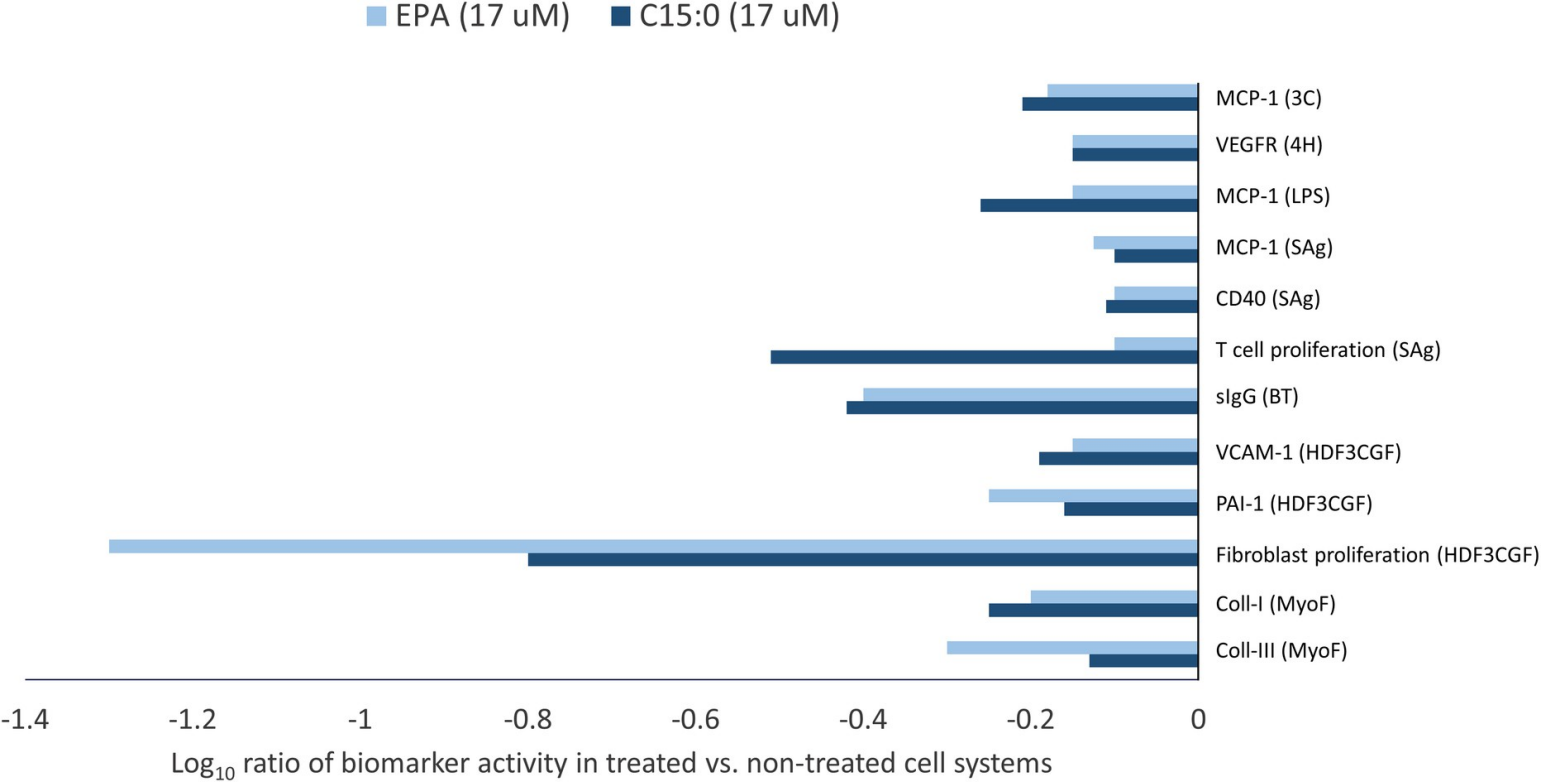
Shared human cell-based activities between C15:0 and EPA. Clinically relevant biomarker activities that were common when comparing C15:0 (FA15^TM^) and EPA at 17 μM. Common biomarker readouts were included when the readout for both profiles was outside the significance envelope with an effect size > 20% (|log_10_ *ratio*| > 0.1) in the same direction.

At EPA’s top non-cytotoxic concentration of 17 μM, comparative overlay analysis of C15:0 (FA15^TM^) and EPA identified 12 common and 35 differentiating activities. Common activities between C15:0 and EPA, all of which involved lowered biomarker activities compared to non-treated control systems, are summarized in Fig 4.

Of the 12 common activities detected between C15:0 and EPA, two were related to inflammation, both of which were anti-inflammatory activities (lower VCAM and MCP-1) compared to non-treated control systems. There were also two common activities related to immunomodulation, both which were down-regulated activities (lower secreted IgG and CD40). Three activities shared between C15:0 and EPA were related to fibrosis, all of which (collagen I, collagen III and PAI-I) were lower compared to non-treated control systems. An additional biomarker activity shared between C15:0 and EPA was lower VEGFR2.

**Fig 5:**
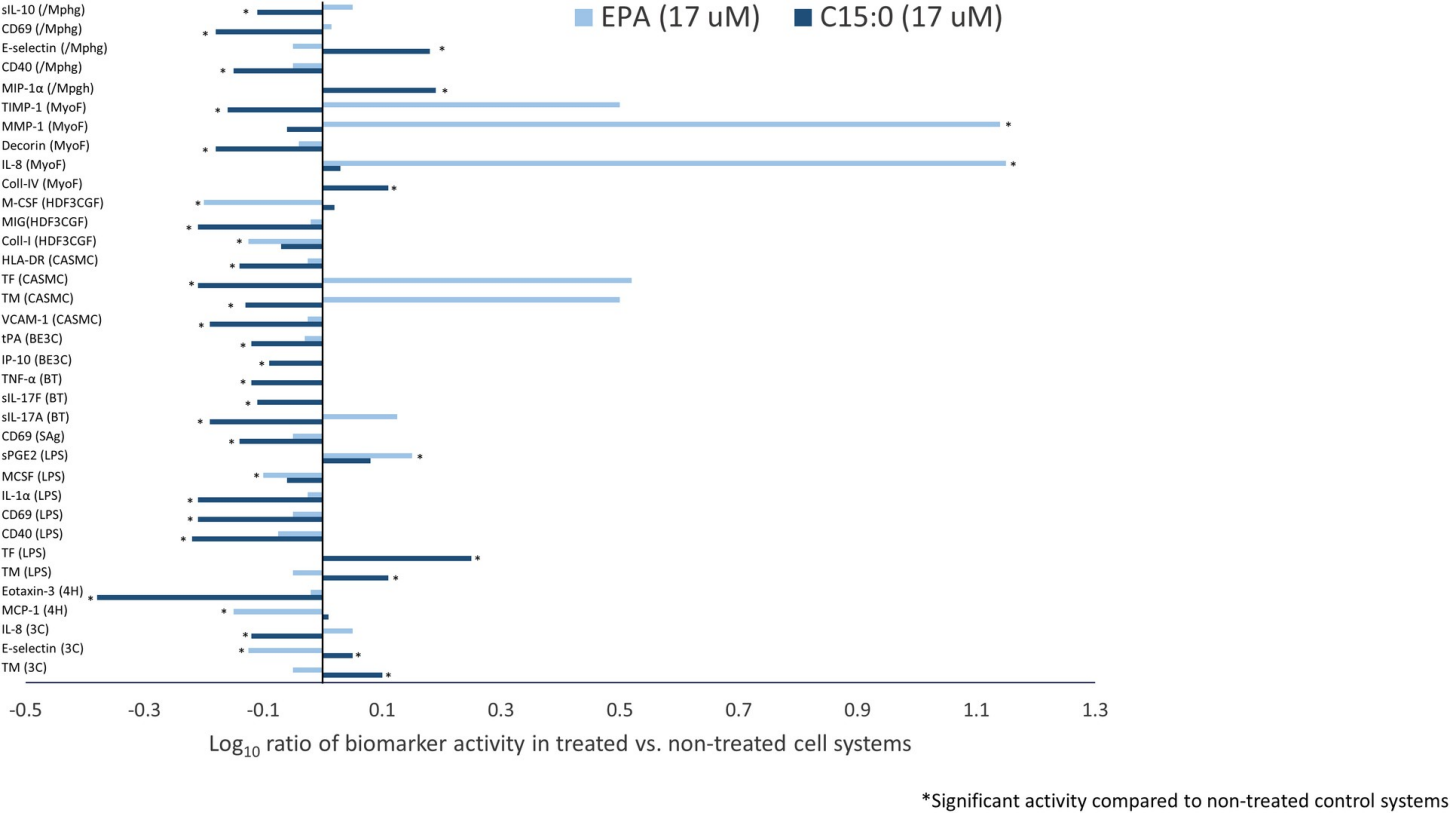
Differentiated human cell-based activities between C15:0 and EPA. Clinically relevant biomarker activities that were differentiating when comparing C15:0 (FA15^TM^) and EPA at 17 μM. Different biomarker readouts were included when the readout for one compound was outside the significance envelope, and the other was not or was outside the significance envelope in the opposite direction.

In contrast to 12 shared activities, there were 35 differentiating activities when comparing C15:0 and EPA (Fig 5). Nine differentiating activities were related to inflammation. Specifically, C15:0, but not EPA, effectively lowered TNFα, P-selectin, eotaxin-3, IP-10, MIG, and IL-1α.

There were an additional five differentiated activities between C15:0 and EPA related to immunomodulation, four of which were down-regulated activities (lower CD69, HLA-DR, IL-17A, and IL-17F) present with C15:0 but not EPA. There were five differentiated biomarker activities between C15:0 and EPA related to fibrosis, of which three (tPA, TIMP-1 and decorin) were lower with C15:0 but not EPA compared to non-treated control systems.

### Top database search results for C15:0

Including all four C15:0 concentrations tested across 12 human cell systems and 148 clinically relevant biomarkers, an unsupervised search for mathematically similar compound profiles from the BioMAP Reference Database was conducted. Of more than 4,500 compounds included in this search, six therapeutic compounds met the threshold (Pearson score ≥ 0.7) for mechanistically relevant similarities to C15:0 (Table 1). At the lowest concentrations, C15:0 matched that of bupropion, a commonly used antidepressant, as well as CP 55,940 a dual cannabinoid receptor 1 and cannabinoid receptor 2 agonist. C15:0 at 5.6 μM also mimicked two antimicrobials, climbazole and clarithromycin. At the highest 50 μM concentration, C15:0 mimicked two anti-cancer drugs, gemcitabine and paclitaxel.

**Table 1:** Top BioMAP reference database matches for C15:0 (pentadecanoic acid). A table of the top similarity matches from an unsupervised search of the BioMAP Reference Database of > 4,500 agents for each concentration of test agent. Profiles are identified as having mechanistically relevant similarity if the Pearson’s correlation coefficient is ≥ 0.7.

C15:0 concentration	Database Match	BioMAP Z- Standard	Pearson’s Score	# of Common Readouts	Mechanism Class
**1.9 μM**	Bupropion (10 μM)	12.481	0.777	148	Norepinephrine-dopamine reuptake inhibitor
	CP 55, 940 (1.1 μM)	11.804	0.753	148	Cannabinoid receptor agonist
**5.6 μM**	Bupropion (30 μM)	12.716	0.784	148	Norepinephrine-dopamine reuptake inhibitor
	Climbazole (10 μM)	12.040	0.762	148	Antimicrobial agent
	Clarithromycin (100 μM)	9.608	0.751	100	Antibiotic agent
**50 μM**	Gemcitibine Hydrochloride (14 nM)	12.279	0.770	148	Chemotherapy agent (nucleoside metabolic inhibitor)
	Paclitaxel Prodrug (120 nM)	11.046	0.742	137	Anti-tumor agent

## Discussion

C15:0 is emerging as an essential fatty acid with pleiotropic and clinically relevant benefits that may help to stem chronic cardiometabolic, liver, and inflammatory diseases [28, 30]. Here, our study demonstrated broad and dose-dependent anti-inflammatory, immunomodulatory and antifibrotic activities of C15:0, including dose dependent activities involving 36 key biomarkers across 10 different human cell systems mimicking various disease states. Overall, these clinically relevant activities were repeated when evaluating two pure C15:0 compounds from different manufacturers.

C15:0 shared twelve anti-inflammatory, immunomodulatory and antifibrotic activities with pure EPA, an omega-3 fatty acid widely used as a dietary supplement, food ingredient, and pharmaceutical. Among these shared activities, both C15:0 and EPA lowered monocyte chemoattractant protein-1 (MCP-1) across three systems mimicking chronic inflammation, cardiovascular disease, and autoimmune disease. MCP-1 is a core chemokine that recruits monocytes and T cells into sites of inflammation, and it plays a central role in the development and severity of numerous diseases, including Alzheimer’s and Parkinson’s disease, epilepsy, multiple sclerosis, cardiovascular disease, stroke, type 2 diabetes, tuberculosis, COVID-19, osteoarthritis, rheumatoid arthritis, and osteoporosis [31]. Demonstrated MCP-1 lowering capabilities of C15:0 is consistent with our prior study, where daily oral C15:0 supplementation for 12 weeks successfully lowered circulating MCP-1 in a high-fat diet induced obese mouse model of type 2 diabetes [28]. These findings warrant further investigations into how C15:0 and EPA may help to manage chronic diseases by lowering MCP-1.

In addition to MCP-1, C15:0 and EPA effectively lowered CD40 and T cell proliferation in a single system relevant to T-cell driven inflammatory conditions. This cell system is used to discover potential therapeutics for autoimmune diseases, including rheumatoid arthritis, psoriasis, and Crohn’s disease, as well as for hematological oncology applications. Although use of EPA has been proposed as a method to manage autoimmune diseases, supportive studies have been primarily limited to fish oil that did not discriminate between the effects of EPA versus other fatty acids [32–36]. Our study is the first to report the potential for C15:0 to manage autoimmune diseases. In humans, higher circulating C15:0 concentrations have been repeatedly linked to a lower risk of metabolic diseases, including type 2 diabetes and nonalcoholic fatty liver disease, and daily C15:0 supplementation can attenuate these conditions *in vivo* [16, 23–25, 28]. Since metabolic disturbances, including those involving nutritional lipids, may also play a role in development of autoimmune diseases, further investigation into how C15:0 may treat metabolic conditions and associated autoimmune diseases are needed [16, 36].

Multiple biomarkers in two human cell systems mimicking chronic inflammation, fibrosis, and stromal biology in tumors were lowered by both C15:0 and EPA. Consistent with these findings, higher circulating C15:0 concentrations have been associated with less severe liver fibrosis among patients with nonalcoholic steatohepatitis [24]. Further, daily C15:0 supplementation in an *in vivo* nonalcoholic steatohepatitis model resulted in less severe liver fibrosis compared to non-treated controls [28]. Similarly, EPA has been shown to attenuate both liver and renal fibrosis [37, 38]. Combined, these studies support the potential use of C15:0 and EPA to manage fibrotic and tumor-related diseases.

While C15:0 and EPA shared 12 common activities, our studies also demonstrated 35 differences between these two fatty acids, including substantially broader anti-inflammatory, immunomodulatory and antifibrotic activities caused by C15:0 that were not present with EPA. Of the 11 cell systems in which C15:0 had disease-attenuating properties that were not present with EPA, three were relevant to atherosclerosis, vascular inflammation, and restenosis, as well as two others relevant to asthma, allergies, and metabolic diseases. Examples of biomarkers lowered by C15:0 and not EPA in these systems included sIL-10, CD69, HLA-DR, TNF-α, IL-17F, IL-17A, and IL-1α. Given these findings, further studies are warranted to evaluate if C15:0’s broader therapeutic activities at the cellular level translate to broader health benefits to individuals and populations compared to EPA and other omega-3s fatty acids.

Demonstrated mechanisms of actions for C15:0 and EPA may help explain both their shared and differentiated activities. C15:0 and EPA are endogenous peroxisome proliferator activated receptor (PPAR) agonists, including PPAR alpha and delta; their roles as PPAR agonists can explain their shared anti-inflammatory and antifibrotic activities [28, 39]. Additionally, C15:0 and EPA have been shown to target the AMP-activated protein kinase (AMPK) pathway, which modulates glucose metabolism [23, 40, 41], as well as inhibit histone deacetylase (HDAC), a proposed means of treating cancer by stemming cancer cell proliferation [26, 42]. While C15:0 and EPA share several key mechanisms of action, they appear to have opposing effects related to MAPK and JAK-STAT signaling, which use oxidative stress to elicit cytokine and inflammatory processes. Specifically, polyunsaturated fatty acids, like EPA, induce MAP and JAK-STAT signaling, while C15:0 inhibits these pro-inflammatory pathways [29, 43, 44]. JAK-STAT inhibitors have been proposed as promising therapeutics to inhibit cytokines and treat numerous inflammatory and autoimmune diseases, and this key mechanism of C15:0 may explain why it had broader anti-inflammatory effects in our study compared to EPA [45].

Associated with C15:0’s protection against oxidative stress compared to EPA, is its functional role in the cell membrane. While C15:0 was safe to cells across all systems and tested concentrations, EPA at 50 μM was toxic to cells in four of the 12 systems. Polyunsaturated fatty acids, including EPA, have multiple double bonds, which make them oils at room temperature and more susceptible to lipid peroxidation, including in the body. In turn, lipid peroxidation has been recognized as a driver for numerous chronic diseases, as well as aging-related degradation [46]. While EPA has downstream antioxidant properties, EPA supplementation in humans can also result in increased lipid peroxidation, especially at doses higher than 2.5 grams per day and among people who are older [47–49]. In comparison, C15:0’s cellular stability at high concentrations is consistent with its role as a saturated fatty acid, which contains no double bonds and is resistant to lipid peroxidation; the beneficial role of higher fatty acid saturation and lowered lipid peroxidation in cell membranes have been attributed to healthier aging and longevity [50].

When assessing clinically relevant and dose-dependent activities of C15:0 and over 4,500 additional compounds, our study demonstrated common cell-based phenotypic profiles between C15:0 and therapeutics for mood disorders, infections, and cancer, based on concentration. At lower concentrations (1.9 and 5.6 μM), C15:0 human cell-based activities closely matched those of bupropion at 10 and 30 μM, respectively. Bupropion, sold as Wellbutrin^®^, is a dopamine-norepinephrine reuptake inhibitor and commonly used antidepressant that is considered safe, well tolerated, and does not result in weight gain [51]. Specific conditions managed by bupropion include major depressive disorder and seasonal affective disorder, and it has shown promise as a non-nicotine agent that promotes smoking cessation in clinical trials [51]. Bupropion is a pill that is typically taken 2–3 times a day in doses ranging from 100–150 milligrams. Based on human pharmacokinetic data with pure free fatty acid C15:0, approximate doses of 19 to 56 mg is expected to achieve circulating C15:0 concentrations with activities similar to bupropion [52].

Poor diet quality has been linked to risk of developing depression, and countries which have diets that contain more essential fatty acids have lower incidence of depression compared to those with low levels of essential fatty acids [53, 54]. These findings suggest that supplementing essential fatty acids, such as omega-3s, could help curb depression as well as many other disorders, including improving cognitive function in patients with schizophrenia and attenuating affective mood disorder [55]. Given this study’s demonstration of common cell-based profiles between C15:0 and bupropion, this study is the first to support this emerging essential fatty acid as a potential means to prevent and manage depression and mood disorders. As such, clinical trials are warranted to evaluate C15:0 as a means to support mental health.

In addition to bupropion, C15:0 at 1.9 μM shared cell-based disease-attenuating activities with CP 55,940, a cannabinoid receptor 1 and 2 agonist. Cannabinoid receptor agonists have been shown to attenuate mood and anxiety disorders, as well as potential treatments for to a wide variety of conditions [56, 57]. While C15:0 at 1.9 μM had human cell-based activities similar to CP 55,940, our prior studies showed that C15:0 up to 20 μM did not have significant direct cannabinoid receptor agonist activities compared to CP 55,940 [28]. As such, further studies are needed to better understand shared cell-based and clinically relevant activities between C15:0 and cannabinoids, understanding that C15:0 itself is not a directly potent cannabinoid receptor agonist.

At 5.6 μM, C15:0 had cell-based activities that closely matched those of two antimicrobials, climbazole and clarithromycin. Climbazole is an imidazole antifungal agent used as a topical treatment for skin infections, dandruff and eczema [58]. Clarithromycin is a broad-spectrum macrolide antibacterial agent widely used for infections caused by *Staphylococcus* and *Streptococcus* species as well as atypical pneumonia [59]. It has been previously demonstrated that C15:0 has antimicrobial activities, including against fungi (*Candida* and *Aspergillus* species) and bacteria (*Staphylococcus aureus*, *Bacillus subtilis*, *Pseudomonas aeruginosa*, *Escherichia coli*, and *Proteus vulgaris*) [60]. Due to these properties, including preventing growth of *Candida albicans* and *Klebsiella pneumoniae*, C15:0 has been proposed as an effective means to prevent severe infections caused by polymicrobial biofilms [61]. Further studies are needed to evaluate C15:0’s role in preventing and treating systemic fungal and bacterial infections.

At the highest dose tested (50 μM), C15:0 mimicked two anti-cancer therapeutics, gemcitabine hydrochloride and paclitaxel prodrug, indicating these compounds share mechanistically relevant similarity, including antiproliferative properties. Gemcitabine is a broad-spectrum antimetabolite and deoxycytidine analogue that has antineoplastic activity [62]. It is used as a broad chemotherapy treatment for breast cancer, non-small cell lung cancer, ovarian cancer, and pancreatic cancer; in some cases, use of gemcitabine with paclitaxel can be more effective [62, 63]. Consistent with this study’s findings, including demonstrated broad antiproliferative properties of C15:0 and phenotypic, mechanistic matching with two anti-cancer therapeutics, C15:0 has been recently shown to suppress breast cancer cells and inhibit dysregulation of histone deacetylase 6, a driver for epigenetic-based cancers [26, 29]. Given this study’s added support of C15:0 as an anti-cancer therapeutic, *in vivo* studies are warranted to further evaluate C15:0’s efficacy and safety at higher doses against a variety of cancers.

While we demonstrated the repeatable safety and clinically relevant, dose-response activities of C15:0, this study was limited to evaluating the effects of C15:0 on primary human cell systems mimicking inflammatory, autoimmune, proliferative, and fibrotic disease states, including immune, pulmonary, vascular, and epithelial cells. Our use of twelve primary human cell lines representing multiple organ systems and disease states, along with non-treated control systems, enabled more robust conclusions compared to using a single, non-primary cell line; that said, these cell-based studies are intended to predict outcomes that need to be tested in *in vivo* studies and clinical trials, especially those related to mood disorders and cancer. While the current study demonstrated overall similarities in clinically relevant biomarkers when comparing C15:0 products from two different sources, five (3%) of the 148 biomarkers were different. Future studies may want to evaluate whether these differences were due to true differences in the two C15:0 compounds or variation in the BioMAP readouts. Additionally, this study was limited to comparisons of C15:0 with EPA and no other omega-3 fatty acids, including DHA and alpha linolenic acid. Finally, this study was limited in its evaluation of free fatty acid C15:0 and did not include other lipid species containing C15:0, including C15:0 metabolites. In food sources, including dairy fats, C15:0 is naturally a component of triglycerides that are broken down by digestive enzymes into free fatty acids, monoglycerides and diacylglycerides before being absorbed by the gut [60]. Once absorbed, dietary fatty acids are readily integrated into numerous lipid species. Further, C15:0 can be elongated to C17:0, as well as metabolized down to propionic acid [24, 61]. Expanded evaluation of C15:0 metabolites using cell-based and *in vivo* studies could provide a more comprehensive assessment of C15:0 and its broader role in maintaining physiological health.

## Conclusion

Demonstrated broad and beneficial activities of C15:0, including many that were improved upon a leading omega-3, support C15:0’s role as an emerging essential fatty acid relevant to both physical and mental health. This study built on the existing literature of C15:0’s anti-cancer and antimicrobial properties, as well as being the first to demonstrate the potential for C15:0 to help manage depression and autoimmune diseases. Given the growing and repeated evidence of C15:0 as a beneficial odd-chain saturated fatty acid, there is a need to reevaluate current nutritional guidelines that continue to recommend lowered intake of all dietary saturated fats.

## Supporting information

S1 Table Description of 12 primary human cell systems included in the BioMAP^®^ Diversity Plus system. (PDF)

S2 Table Eurofins DiscoverX BioMAP data on pentadecanoic acid (C15:0) and eicosapentaenoic acid (EPA). (XLSX)

## Abbreviations

α-IGM: immunoglobulin M-alpha
α-SMA: alpha smooth muscle actin
bFGF: basic fibroblast growth factor
C15:0: pentadecanoic acid
CD14+: cluster of differentiation 14
CD19+: cluster of differentiation 19
CD40: cluster of differentiation 40
CD69: cluster of differentiation 69
CD90: cluster of differentiation 90
Col-I: collagen I
Col-III: collagen III
Col-IV: collagen IV
CR: common readouts
DHA: docosahexaenoic acid
DMSO: dimethyl sulfoxide
EGF: epidermal growth factor
EGFR: epidermal growth factor receptor
ELISA: enzyme-linked immunoassay
EPA: eicosapentaenoic acid
HLA-DR: human leukocyte antigen–DR isotype
ICAM: intracellular adhesion molecule 1
IFNγ: interferon gamma
IL-1α: interleukin 1 alpha
IL-1β: interleukin 1 beta
IL-2: interleukin 2
IL-4: interleukin 4
IL-6: interleukin 6
IL-8: interleukin 8
IP-10: interferon gamma-induced protein 10
I-TAC: interferon-inducible T-cell alpha chemoattractant
HTRF: homogenous time resolved fluorescence
HUVEC: human umbilical vein endothelial cells
LDLR: low density lipoprotein receptor
LPS: lipopolysaccharide
MCP-1: monocyte chemoattractant protein-1
MIG: membrane immunoglobulin
MIP1α: macrophage inflammatory protein 1 alpha
MMP-1: matrix metalloproteinase-1
MMP-3: matrix metalloproteinase-3
MMP-9: matrix metalloproteinase-9
PAI-1: plasminogen activator inhibitor 1
PBMC: peripheral blood mononuclear cell
PBS: phosphate buffered saline
PDGF-BB: platelet derived growth factor-BB
sPGE2: secreted prostaglandin E_2_
sTNFα: secreted tumor necrosis factor alpha
SAA: serum amyloid A
SRB: sulforhodamine B
TCR: T cell receptor
TF: tissue factor
TGFβ: transforming growth factor beta
TIMP-1: tissue inhibitor matrix metalloproteinase 1
TIMIP-2: tissue inhibitor matrix metalloproteinase 2
TLR2: toll-like receptor 2
TLR4: toll-like receptor 4
TM: trabecular meshwork
TNFα: tumor necrosis factor alpha
VCAM: vascular cell adhesion molecule 1
uPAR: urokinase plasminogen activator surface receptor
uPA: urokinase plasminogen activator
tPA: tissue plasminogen activator
VEGFR-2: vascular endothelial growth factor receptor 2
