# Supplementary material for: Broader and safer clinically-relevant activities of pentadecanoic acid compared to omega-3: Evaluation of an emerging essential fatty acid across twelve primary human cell-based disease systems
Source: PLoS One. 2022 May 26;17(5):e0268778. doi: 10.1371/journal.pone.0268778 (PMC9135213; doi:10.1371/journal.pone.0268778)
Supplement: S1 Table — (PDF) [file pone.0268778.s001.pdf]

Table S1. Description of the 12 primary human cell systems included in the BioMAP® Diversity Plus system (from Eurofins/DiscoverX)

System	Icon	Cell Type	Stimulation	Disease Relevance	Biomarker Readouts
3C	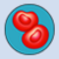	Venular endothelial cells	IL-1β, TNFα and IFNγ	Cardiovascular Disease, Chronic Inflammation	MCP-1, VCAM-1, TM, TF, ICAM-1, E-selectin, uPAR, IL-8, MIG, HLA-DR, Proliferation, SRB
4H	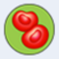	Venular endothelial cells	IL-4 and histamine	Asthma, Allergy, Autoimmunity	MCP-1, Eotaxin-3, VCAM-1, P-selectin, uPAR, SRB, VEGFRII
LPS	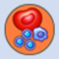	Peripheral blood mononuclear cells + Venular endothelial cells	TLR4 ligand	Cardiovascular Disease, Chronic Inflammation	MCP-1, VCAM-1, TM, TF, CD40, E-selectin, CD69, IL-8, IL-1α, M-CSF, sPGE$_2$, SRB, sTNFα
SAg	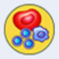	Peripheral blood mononuclear cells + Venular endothelial cells	TCR ligands (1x)	Autoimmune Disease, Chronic Inflammation	MCP-1, CD38, CD40, E-selectin, CD69, IL-8, MIG, PBMC Cytotoxicity, Proliferation, SRB
BT	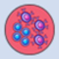	Peripheral blood mononuclear cells + B cells	α-IgM and TCR ligands (0.001X)	Asthma, Allergy, Oncology, Autoimmunity	B cell Proliferation, PBMC Cytotoxicity, Secreted IgG, sIL-17A, sIL-17F, sIL-2, sIL-6, sTNFα
BF4T	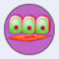	Bronchial epithelial cells + Dermal fibroblasts	TNFα and IL-4	Asthma, Allergy, Fibrosis, Lung Inflammation	MCP-1, Eotaxin-3, VCAM-1, ICAM-1, CD90, IL-8, IL-1α, Keratin 8/18, MMP-1, MMP-3, MMP-9, PAI-1, SRB, tPA, uPA
BE3C	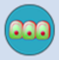	Bronchial epithelial cells	IL-1β, TNFα and IFNγ	Lung Inflammation, COPD	ICAM-1, uPAR, IP-10, I-TAC, IL-8, MIG, EGFR, HLA-DR, IL-1α, Keratin 8/18, MMP-1, MMP-9, PAI-1, SRB, tPA, uPA
CASM3C	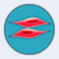	Coronary artery smooth muscle cells	IL-1β, TNFα and IFNγ	Cardiovascular Inflammation, Restenosis	MCP-1, VCAM-1, TM, TF, uPAR, IL-8, MIG, HLA-DR, IL-6, LDLR, M-CSF, PAI-1, Proliferation, SAA, SRB
HDF3CGF	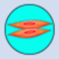	Dermal fibroblasts	IL-1β, TNFα, IFNγ, EGF, bFGF and PDGF-BB	Fibrosis, Chronic Inflammation	MCP-1, VCAM-1, ICAM-1, Collagen I, Collagen III, IP-10, I-TAC, IL-8, MIG, EGFR, M-CSF, MMP-1, PAI-1, Proliferation_72hr, SRB, TIMP-1, TIMP-2
KF3CT	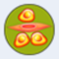	Keratinocytes + Dermal fibroblasts	IL-1β, TNFα, IFNγ and TGFβ	Psoriasis, Dermatitis, Skin Biology	MCP-1, ICAM-1, IP-10, IL-8, MIG, IL-1α, MMP-9, PAI-1, SRB, TIMP-2, uPA
MyoF	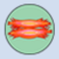	Lung fibroblasts	TNFα and TGFβ	Fibrosis, Chronic Inflammation, Wound Healing, Matrix Remodeling	α-SM Actin, bFGF, VCAM-1, Collagen-I, Collagen-III, Collagen-IV, IL-8, Decorin, MMP-1, PAI-1, TIMP-1, SRB
/Mphg	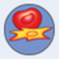	Venular endothelial cells + Macrophages	TLR2 ligand	Cardiovascular Inflammation, Restenosis, Chronic Inflammation	MCP-1, MIP-1α, VCAM-1, CD40, E-selectin, CD69, IL-8, IL-1α, M-CSF, sIL-10, SRB, SRB-Mphg
